# Contemporary Approach to the Porosity of Dental Materials and Methods of Its Measurement

**DOI:** 10.3390/ijms22168903

**Published:** 2021-08-18

**Authors:** Katarzyna Sarna-Boś, Kamil Skic, Jarosław Sobieszczański, Patrycja Boguta, Renata Chałas

**Affiliations:** 1 Department of Dental Prosthetics, Medical University of Lublin, Chodźki 6, 20-093 Lublin, Poland; 2 Institute of Agrophysics, Polish Academy of Sciences, Doświadczalna 4, 20-290 Lublin, Poland; k.skic@ipan.lublin.pl (K.S.); p.boguta@ipan.lublin.pl (P.B.); 3 Preclinical Dentistry Lab, Medical University of Lublin, Chodźki 6, 20-093 Lublin, Poland; jaroslawsobieszczanski@umlub.pl; 4 Department of Oral Medicine, Medical University of Lublin, Chodźki 6, 20-093 Lublin, Poland; renata.chalas@umlub.pl

**Keywords:** porosity, voids, dental materials, dental composite, ceramics, titanium

## Abstract

Porosity is an important parameter for characterizing the microstructure of solids that corresponds to the volume of the void space, which may contain fluid or air, over the total volume of the material. Many materials of natural and technically manufactured origin have a large number of voids in their internal structure, relatively small in size, compared to the characteristic dimensions of the body itself. Thus, porosity is an important feature of industrial materials, but also of biological ones. The porous structure affects a number of material properties, such as sorption capacity, as well as mechanical, thermal, and electrical properties. Porosity of materials is an important factor in research on biomaterials. The most popular materials used to rebuild damaged tooth tissues are composites and ceramics, whilst titanium alloys are used in the production of implants that replace the tooth root. Research indicates that the most comprehensive approach to examining such materials should involve an analysis using several complementary methods covering the widest possible range of pore sizes. In addition to the constantly observed increase in the resolution capabilities of devices, the development of computational models and algorithms improving the quality of the measurement signal remains a big challenge.

## 1. Introduction

**Figure 1:**
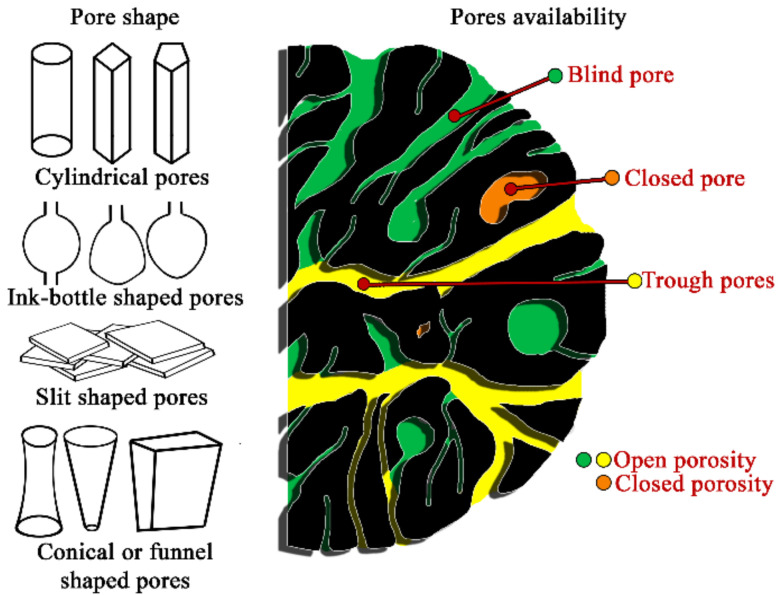
Scheme of pores classifications, according to pores geometrical shape and availability.

Porosity is an important parameter for characterizing the microstructure of solids that corresponds to the volume of the void space, which may contain fluid or air, over the total volume of the material. According to accessibility, the pore system can be divided into two main categories—closed and open porosity. Closed porosity, or residual porosity, refers to pores that have no communication to the surroundings. This type of porosity is not associated with adsorption and permeability, but it influences the mechanical properties of solid materials [1]. On the opposite, pores that have a continuous channel of communication to the outer surface (blind and interconnected pores) create an open porosity, in which the flow of liquids and gases and the accompanying phenomena of heat exchange, filtration, diffusion, sorption, and chemical reactions take place. For this reason, the volume of the open pores is often called the active volume of the pores (or the effective volume). Additionally, the pore system can be clarified according to pore size, shape, functions, or other distinguishing features. Examples of main pore classifications are shown in Figure 1 and Table 1.

Many materials of natural and technically manufactured origin have a large number of voids in their internal structure, relatively small in size, compared to the characteristic dimensions of the body itself [1]. Thus, porosity is an important feature of industrial materials, but also of biological ones. Scientists conducting research related to a living organism are constantly looking for ideal materials that imitate human tissues as much as possible. It is no different in dentistry. Despite many breakthrough achievements, there is still no product as long-lasting as the structure of the tooth itself. The most popular materials used to rebuild damaged tooth tissues are composites and ceramics, whilst titanium alloys are used in the production of implants that replace the tooth root. The porosity of these materials is an important feature influencing their properties. The porous structure affects a number of material properties, such as sorption capacity, as well as mechanical, thermal, and electrical properties. This fact is particularly important in dental biomaterials, the rapid, remarkable development of which has taken place in recent years.

Therefore, the study aims the porosity aspect of the most commonly used dental materials and its assessment methods.

## 2. Porosity of Dental Composite

Resin-based dental composite materials, currently used in dentistry (also referred to as “dental composite” or “dental composite resin” or “dental resin composite” or “dental polymer composite” in the literature) [2], are known for their excellent aesthetic and mechanical properties. These materials are composed of an active organic resin matrix and an inactive, inorganic/organic filler [3]. The organic resin matrix phase is made from a mixture of multifunctional monomers and light-sensitive initiators, while the inorganic/organic filler phase contains micro/nano-sized fillers, which are mainly used as reinforcement [4].

It should be stated, objectively, that the composites, used for the fillings, exhibit some porosity. It depends on many parameters. Depending on the research, the estimated amount of porosity in composites varies and falls mainly in the range of 0.5–4% of the volume [5,6,7]. This inconsistency may be due to the way of working with the material, the properties of the material. Additional factors that influence the porosity of composites are the manufacturing processes, but the impact of this has not yet been thoroughly investigated.

The porosity of composites is related to incomplete monomer conversion to polymers, as well as the presence of oxygen. In addition, oxygen also plays an important role in inhibiting polymerization by binding to free radicals. Therefore, testing the porosity of composites seems to be extremely useful, as the presence of oxygen bubbles in the product may lower the mechanical properties of the material. For example, it has been shown that reducing the porosity of a resin composite from 4.1 to 0.4% increases its traction resistance by 11.5% [8]. An increase in the porosity of the composite material in the range of 1.5–3% by volume reduces the compressive strength and the compressive fatigue limit, estimated at 30–50%, and pores can be considered as critical defects, related to cracking of the restoration [8].

Moreover, the presence of porosities, resulting from the presence of oxygen, has a potential influence on biocompatibility [9]. The porosity of the composites also promotes build-up and retention of dental plaque. Large surface pores can contribute to secondary caries if they are along the critical border of the tooth and filling, but the size threshold value is debatable [10].

The porosity of the composite material may also contribute to the increase of the water absorption capacity and as a result of the diffusion of water molecules into the interior of the composite, morphological voids may be formed [11]. The presence of these spaces leads to translucent zones, which may later become dull or even stained. This has a huge impact on the aesthetics of the filling. The amount of porosity correlates with the increase in water sorption, and thus facilitates hygroscopic/hydrolytic activities, such as swelling (separation of polymer chains), hydrolysis of polymer chain components, and silane degradation between the filler and the composite matrix [12,13].

The porosity of composites is also influenced by other parameters, such as the type of polymerization [14] and the diameter of the applicator, as well as the method of fixing the restoration [15] or the viscosity of the material [16]. A study by Balthazard et al. [17] showed that the lower the viscosity of the material, the higher the internal porosity. Since the intrinsic porosity of the material cannot be controlled or modified by a dentist, strict clinical protocols remain the best way to reduce the porosity of composites [17].

There are many studies on porosity in polymerized composite samples [5,6,7], while few studies have been conducted with non-polymerized material. The studies by Nilsen et al. [18] showed that there are pores in the unpolymerized composite samples, but there was no direct linear correlation with the amount of porosity of the final polymerized restoration. Heterogeneity in the distribution of pores, present in the unpolymerized composite material, was also demonstrated. In the case of restorations made of universal composites, low porosity visible in the intact composite, coupled with the variability in the porosity size of the final restoration, suggests that the manipulation and handling of these materials during work generates pores and voids in the material. In contrast to flowable composites, where a small variation in the size of the porosity was observed. However, most of the pores are located along the contact zone between the material and the tooth wall for all tested materials [18].

The quality and durability of the tooth restoration is, thus, directly related to the degree of porosity of the composite. The production of composite fillings without pores and voids is difficult, regardless of the application technique and material used [17]. From a mechanical point of view, the pores are defects in the material. However, there is no direct clinical evidence on how the amount of porosity can influence the clinical performance of composite restorations [19].

## 3. Porosity of Dental Ceramics

Dental ceramics are materials commonly used in dentistry for making permanent restorations. There are various types of ceramics available on the market. All-ceramic and ceramic-like restorative materials can be categorised into three groups: (1) glass-matrix ceramics, (2) polycrystalline ceramics, and (3) resin-matrix ceramics. Glass-matrix ceramics” are nonmetallic inorganic ceramic materials that contain a glass phase, while “polycrystalline ceramics” are defined as nonmetallic inorganic ceramic materials that do not contain glass, but only a crystalline phase. In the third group—“resin-matrix ceramics” are included materials that have a polymer matrix, containing predominantly inorganic refractory compounds. In addition to perfect aesthetics, these materials are characterized by high strength, good color stability, high abrasion resistance, and high biocompatibility [20,21]. They are characterized by the presence of traces of pores and negligible water absorption, which is very desirable. Currently, many all-ceramic systems are available for use in permanent restoration prosthetics.

Feldspar ceramics consist of significant amount of feldspar, quartz, and kaolin. Feldspar is a greyish crystalline mineral that can be found in rocks rich with iron and mica. Quartz or silica is the matrix component (55–65%) responsible for the translucency of the restoration. As it is not a strong material, 20–25% alumina is added as a reinforcing component. Kaolin is a hydrated aluminium silicate that is used in a limited amount (4%), as it has opaque properties, unlike the human teeth which are translucent [21,22]. This porcelain corresponds aesthetically to natural teeth, thanks to the moderate translucency and similar color. However, it is a brittle material with low strength, in the range of flexural strength 60–110 MPa [Table 2], which means that it is limited to making only one-point restorations. It is also characterized by high hardness, which is why it has an abrasive effect on the opposing teeth.

The basic component of the leucite-reinforced glass ceramic is feldspathic porcelain, consisting of 63% SiO_2_, 19% Al_2_O_3_, 11% K_2_O, 4% Na_2_O, and traces of other oxides. Leucite crystals are added to the aluminum oxide with a grain size of 1 to 5 μm [23]. This material is manufactured using a process known as heat pressing, which is performed in an investment mold. This mold is filled with the plasticized ceramic, thus avoiding the sintering process and the subsequent pore formation. This ceramic undergoes dispersion strengthening through the guided crystallization of leucite [24]. It is translucent, which gives a satisfactory aesthetic effect. Strengthening with leucite resulted in a twice increase in strength, compared to traditional feldspar ceramics, thanks to the fact that the crystals block the spread of microcracks.

Lithium disilicate ceramics contain 60% of longitudinal lithium disilicate crystals that reflect and refract light, thanks to which the translucency of the material is maintained [25]. These ceramics claimed to be highly translucent, due to the optical compatibility between the glassy matrix and the crystalline phase, which minimizes internal scattering of the light as it passes through the material [24]. They are supplemented with tiny lithium orthophosphate crystals. The flexural strength of this material is 350 MPa [Table 2] and is three times higher than that of leucite ceramics, which is related to the densely packed grains in the composition and the elongated shape of the crystals, which inhibit the spread of microcracks. Despite its high strength, these ceramics are characterized by reduced hardness, which is close to the enamel tissue, and therefore is not a traumatic factor for the opposing teeth [25].

Alumina ceramics infiltrated with glass contain 82% alumina, 12% lanthanum oxide, 4.5% silicon oxide, and 0.8% calcium oxide. The strength of this material is in the range of 350–600 MPa [Table 2], it is characterized by a dense structure of the material and high hardness of 11.5 GPa [22]. It contains a characteristic amplification mechanism, called the bridging effect. When there is damage, it does not develop, due to the presence of hard, compact grains in the structure, which are an obstacle to the subsequent fracture, because it has to bypass each grain one by one; as a result, it loses its energy and stops.

Pure alumina ceramic consists of 99.9% aluminum oxide crystals, with a particle size of 4 μm, it is used in CAD/CAM technology. It has a high strength of approx. 700 MPa and high density. This porcelain does not contain a vitreous phase, which results in reduced translucency. In the case of heavily discolored pillars, no translucency is desired to mask the disadvantageous feature [25].

The 99.9% zirconium oxide ceramic consists of yttrium-reinforced zirconia crystals. It occurs in 3 forms of crystal lattice, i.e., monoclinic, tetragonal, and cubic. It is the most durable material of all types of ceramics. It has a high strength of 840–1200 MPa [Table 2] and the ability to block microcracks by transforming the tetragonal phase into a monoclinic phase, which is associated with an increase in particle size and the elimination of damage [26].

Among the presented ceramics, glass ceramics is one of the most popular, due to its good marginal fit, good mechanical properties, and low porosity, compared to conventional feldspar porcelain [27,28]. It has been shown that the microstructure, and thus, the mechanical properties of glass ceramics, can be modified by changing the heat treatment to which it is subjected [29]. The differences in injection temperatures affect the porosity and mechanical properties of the material [20]. The problems with furnace calibrations can lead to a high-volume fraction of porosity and large pore sizes in the material, which may compromise the mechanical properties. Increases in pore size and volume fraction may occur spontaneously in high temperature sintered ceramics, due to gas expansion inside the pores caused by pore growth and coalescence [30]. The gas trapped (dissolved) in the glassy matrix can diffuse through it, as the viscosity drops, due to the increase in temperature, leading to the formation (precipitation) and growth of pores in the microstructure [31].

Feldspar ceramics, on the other hand, are the oldest known ceramics in prosthetics. In addition to high aesthetics, this ceramic has a high resistance to wear and compressive loads and is biocompatible [32,33]. However, its mechanical strength is lower than other types of ceramics [32].

On the one hand, the porosity of dental porcelain must be minimized, to obtain the best optical appearance and strength, as the pores scatter light, reducing translucency, and can act as high stress crack initiators, lowering tensile and shear strength [34]. On the other hand, however, restorations made of this ceramic require the preparation of the inner surface, increasing its roughness, in order to bond with the tooth structure with adhesive cements. As the number of adults requiring orthodontic treatment increases, there is also a need for a reliable method of bonding brackets to ceramic surfaces [35]. Both processes require micro-roughing and surface cleaning to ensure adequate retention [36]. Etching with hydrofluoric acid (HF) or sandblasting with alumina particles, or sometimes a combination of both, are among the most common surface treatments of silica-based ceramics [37] and are a key element of the clinical success of cemented ceramic restorations, as well as the bonding of orthodontic brackets to ceramic surfaces [35]. In Valian and Moravej-Salehi’s research [37], SEM images, after the application of HF acid to the surface of feldspar porcelain, showed small and large microporosity, as a three-dimensional network of channels and voids, increasing the surface micro-roughness. These porosities had a tunneling, micro-retention pattern, resembling a honeycomb pattern [37]. This is consistent with the results of studies by Borges et al. [38], Bottino et al. [39], and Kukiattrakoon and Thammasitboon [40]. SEM images of sandblasted surfaces in Valian and Moravej-Salehi’s studies [37] revealed increased surface irregularity, micro-roughness, and porosity with sharper peaks, but shallower than those treated with HF acid alone [37]. During sandblasting, the surface of the ceramic is mechanically bombarded by pressurized aluminum oxide particles. In some studies, such as Amin Salehi et al. [41], increasing the sanding time of feldspar porcelain increased the surface roughness but did not increase the bond strength. Kern et al. [42] reported in their 2009 studies that by increasing the sandblasting pressure with zirconium oxide, the surface roughness increased, but the bond strength was not increased. Thus, in the presence of an appropriate bond strength, there is no need to increase the surface roughness, as the roughness of the porcelain can reduce the strength of the porcelain restoration [43] and even its bending strength. In addition, rough porcelain, following the removal of orthodontic brackets, may increase plaque build-up, which causes gingivitis and unwanted soft tissue responses [43]. Too much roughness of the surface can affect the gloss and color of the porcelain and lead to discoloration. The aesthetics of porcelain may deteriorate [40].

Most of the reduction in porosity takes place in the sintering process, which aims to produce a possibly non-porous solid substance [34]. According to some researchers, this reduction in the pore volume depends on the sintering time, temperature, atmosphere, and alloy viscosity [34]. However, minimal porosity is only achieved in a very narrow range of conditions, generally at high temperature and short sintering times. A higher sintering temperature correlates with a higher hardness number. SEM analysis indicates lower porosity in samples sintered at higher temperatures [34].

Crystals present in dental ceramics have isotropic properties that play an important role in modifying their properties such as material hardness, flexural strength, modulus of elasticity, and fracture toughness [44,45]. However, the presence of pores can interfere with stress distribution as they act as stress areas and affect the mechanical properties, favoring mechanical failure.

## 4. Porosity of Titanium and Its Alloys

Titanium, and its alloys showing high specific strength, are materials of choice for the production of orthopedic and dental implants. Properties that determine whether a material is suitable for biomedical implant applications include biocompatibility, bioadhesion, biofunctionality, and corrosion resistance. [46,47]. Titanium is considered to be biocompatible because it has a low electrical conductivity which contributes to the electrochemical oxidation of titanium leading to a thin passive oxide layer. The oxide layer provides high corrosion resistance, which makes titanium a stable material [46]. Titanium is biocompatible, because it is biologically nearly inert and well tolerated by the environment of the human body [48].

The surface morphology of the implant plays a key role in bone contact with an implant and may improve the osseointegration process. To improve the stability of a dental implant, various surface modifications are proposed, to adapt the properties of titanium implants. Modification of the implant surface may improve the bone-implant interaction, but there is not always a clear explanation for this mechanism [48,49]. Traditional titanium structure, used in the manufacture of medical implants and dental care, is non-porous.

Most dental implant materials support cell attachment by providing a suitable area for cell adhesion [50]. It was found that the behavior of bone cells is more influenced by the topography of the implant surface, with which they are in contact, than by the chemistry of the implant material or the method of its processing [51], although these effects are difficult to separate because they are all interrelated [48,50]. Micro- and macro-roughness of surfaces of dental and orthopedic implants plays an important role in improving the response of osteoblasts [52]. Rough surfaces ensure better adhesion of osteoblasts [53], improved cell proliferation, and extracellular matrix formation, which, in turn, improves the osseointegration process and durability of dental implants [54]. The porous surface of the implant has a large surface area, which should allow more cells to attach than the flat surface [48].

In the case of porous titanium, with a properly designed microstructure, bone tissue ingrowth into the pores of the material can be observed, thanks to which the implant-bone connection becomes much stronger. Additional improvement of osseointegration can be obtained by modifying the material with bioactive phases, enabling creation bonding with tissue of a chemical nature. Connection between the bone and the implant is more important for the vascular system than the size of the pores, because it determines both the number and size of blood vessels [55,56].

In the studies of Shibli et al. [57], in histological evaluations, measured the response of human bone tissue to two types of titanium dental implants: laser-made and sand-blasted, as well as acid-etched and mechanically processed under no load conditions. The result indicated that eight weeks after implantation, the contact between the bone and the CAD-CAM implant did not differ significantly, but it was higher than in the case of a machine-processed implant. The rough surface topography of the implant on a micrometer scale improved the osteogenic response in comparison with machined surfaces of dental implants under no-load conditions. The authors attributed their discovery to the surface roughness that was produced using laser techniques, which enhanced the osseointegration process [48,57].

The mechanical properties of an implant are strongly related to its porosity characteristics. The fraction, size, morphology and distribution of the pores in the material are the main parameters influencing the mechanical properties of the porous structure of the implant for orthopedic and dental applications. Therefore, the aim is to produce porous titanium structures in which the percentage of porosity is controlled in order to reduce the stiffness of the implant without adversely affecting its mechanical properties. In general, the mechanical properties of the porous structure decrease with increasing porosity [55]. The mechanical strength of porous titanium decreases dramatically with the increase of porosity, which on the other hand is a necessary condition for the ingrowth of new bone tissues and vascularization [58].

Porous structures are ideal surfaces because they significantly reduce the stiffness of the implant compared to flexible materials [59]. As a result of the improved transfer of stresses between the implant and the bone through porous structures, a greater biological adhesion between the implant and the tissue is obtained [55]. In this way, osseointegration is facilitated as a larger clamping surface is provided [60]. Moreover, the porous biomaterial enables the recruitment and penetration of cells from the surrounding bone tissue into its structure, promoting bone regeneration and vascularisation [55,61]. A study on male Sprague-Dawley rats showed that the biological stabilization of the implant was influenced by the porosity of the titanium implants (25%, 11%, and 3%). After 16 weeks of testing, the concentration of calcium ions increased in proportion to the increase in the percentage of porosity [48,62].

However, the Young’s modulus of the materials used for the production of implants is higher than that of the mineralized tissue [55]. Titanium has a modulus of elasticity (100–110 GPa) much lower than other implantable metals. However, the modulus of elasticity of human bone is even lower. Compact or cortical bone has approximately 17–20 GPa of Young’s modulus, while spongy bone has less than 4 GPa [63]. Thus, the modulus of elasticity of the biomaterial is the key point for the force distribution in the implant area and the stress shield effect [55].

One solution to the problems associated with titanium’s high modulus of elasticity is the use of advanced manufacturing processes, such as additive layer fabrication (ALM), to produce highly porous titanium structures with CP-Ti and Ti-64. These porous structures can be customized to have excellent mechanical properties similar to human bones and are usually designed to facilitate bone ingrowth [46]. Thanks to advanced production, it is also possible to produce more complex porous structures with improved mechanical parameters, potentially matching the modulus of local bone elasticity [48].

In fact, it is recognized that porous titanium structures should combine high porosity (up to 45–80%) and large pore size (150–500 µm), favoring bone growth and implant osseointegration [64]. The presence of microporosity (less than 10 μm) is also recommended. This microporosity increases the surface area of the porous structure, which contributes to greater absorption of bone-inducing proteins, as well as ion exchange (Ca_2_ +/PO_4_^3−^) and the formation of apatite (bone mineral phase) [65] of the titanium porous structures. It is not easy due to the specific properties of this implantable metal [55]. The optimal pore size is probably between 200 and 1000 μm, and roughness and surface chemistry also play an important role in determining the fate of stem cells and osteoblasts [48].

For example, a morphological or chemical mechanism, such as roughening the implant surface, can also modify the surface chemistry of a dental implant [66]. Plasma spraying of various powder particles such as titanium oxide, calcium phosphate and hydroxyapatite was used to coat dental implants [67]. Sandblasting with stiff particles such as alumina, TiO_2_ and ceramics has also been suggested to roughen the surface of a dental implant [68]. Çelen and Özden in 2012 [69] opted for a different, more controlled laser micromachining technique for a commercially pure titanium dental implant [48,69].

The processing of titanium alloys produced by advanced powder production methods such as incremental film fabrication (or 3D printing) and metal injection molding is clearly receiving more attention and is being adopted as an alternative to machining and casting [48].

As these three parameters (porosity volume, pore size and interconnections) affect the extent of cell ingrowth, they should be adjusted depending on the application for which the implant is intended, as well as the function and location of the target bone [55].

## 5. Methods Used to Assess Porosity in Materials

Knowing the porosity of materials is extremely important, but the greatest difficulty is their assessment due to the variety of methods used in the biomaterials chemistry.

**Figure 2:**
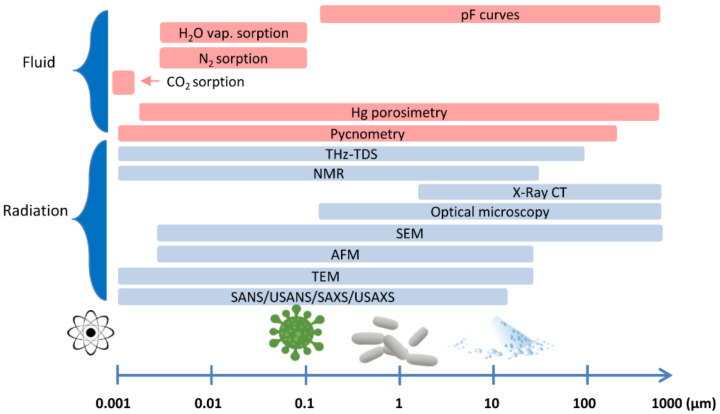
Comparison of the measurement range of different analytical methods used to characterize porosity and pore size distribution. The *X*-axis is represented with a logarithmic scale.

Due to the wide range of pore sizes, there is no universal method for determining the pore size distribution in porous materials [70]. The differentiation of the measuring ranges in the most frequently used methods is shown in Figure 2.

Pore characteristics are usually obtained indirectly from physical measurements or by using digital image processing and analysis, and computed tomography techniques that can provide a more direct way to obtain porosity data. Below, we provide an overview of the most important direct and indirect methods used to assess porosity materials.

Mercury intrusion porosimetry (MIP) is one of the indirect, routine methods that is widely used for determining the parameters of the pore structure in adsorbents, catalysts, ceramics, biomedical, geological and cement-based materials [71,72]. MIP measurements are used to estimate total porosity, to determine characteristic pore size, and to investigate various characteristics of the pore space allow revealing a variety of physical properties of the solid materials [73,74]. The obtained pore size distribution curves can be parameterized with various models (e.g., network, fractal dimension) that allow to estimate pore surface heterogeneity, coherence, tortuosity, permeability, etc. [75,76,77]. A huge advantage of the method is that MIP can measure volumes over a wide range of pore sizes, from approximately 3 nm up to 1000 µm [78]. Additionally, both powders and larger pieces of solids can be tested; however, the latter have been shown to exhibit less variation, resulting in a better representation of the original pore structure [79]. MIP can also be performed in an extrusion mode when data are collected with decreasing pressure steps [80,81]. Analysis usually reveals then hysteresis between intrusion and extrusion cycles which is attributed to variations in the sample saturation process, alterations due to advancing and receding contact angles and mercury entrapped in the pores structure [74,82].

The basic principle of this method is the use of mercury as a non-wetting medium that is forced into the sample according to external pressure. When pressure is applied, the pore structure is gradually filled from the pores with the largest diameters to those with the smallest diameters. Pore size distributions can be determined from the volume of mercury that is intruded at each pressure increment, whereas total porosity is determined from the total intruded volume [83]. The applied intrusion pressure is converted into the pore diameter by using the Washburn equation [84]. In calculation, an assumption is made that the pore system consists of parallel, cylindrical, non-intersecting pores of different diameters which is entirely and equally connected to the outer surface of the material and thus are available to mercury.

The MIP method, apart from its advantages such as the wide range of analyzed pores, short time and simplicity of measurement, reveals some important drawbacks. One of them is that the measurement can only provide a valid estimation of pore structure when each pore is directly accessible to mercury or can be reached by mercury through larger pores [73,85,86]. Therefore, MIP cannot provide information about closed pores that are not connected with the outer surface of materials; hence, the total porosity can be underestimated [82,83]. Another disadvantage is that the MIP does not always reflect the actual pore size distribution. MIP is not a suitable method to characterize materials with the predominance of so-called bottle pores [87]. With such a pore structure, mercury cannot intrude into larger pores until the applied pressure is sufficient to force mercury to go through smaller throats. As a result, the volume of these larger pores is underestimated and counted as the volume of smaller throats [85]. For the same reasons, the pore sizes measured by the MIP method are always smaller than those measured by the imaging methods [88]. Only a few studies considered the effect of the bottle pores on the obtained results [73,76,83,86]. Another source of MIP errors may be the assumption about the constant contact angle, which is usually set for calculation between 130–140° [82]. The contact angle may vary due to the material type, surface roughness and drying technique [78]. MIP characteristics can be also affected by the sample drying process [73] which should be carried out to remove water or other chemicals and to ensure that pores are available for mercury intrusion. While several drying techniques, such as solvent replacement, oven-drying, vacuum-drying, and freeze-drying can be proposed, the application affects a different extent of porosity and pore size distribution [78,89,90]. Finally, there are also reports of the possible deformation of the material microstructure during the mercury intrusion process [91,92,93]. The distorted results can be observed, due to the breaking of the inner pore’s walls [94]. In line with [95], the high pressure can make macropores and fractures compacted, open the pores closed in the initial state, and generate new connecting pores.

Gas adsorption techniques are another indirect method used to determine the pore surface properties and pore size distribution. These techniques use an adsorption phenomenon that could be defined as the process of gas or vapour interaction with the solid surface, causing a change in the component concentration in the interface. The solid phase is then called an adsorbent, the gas or vapour that can adsorb is called an adsorptive, and the fluid in the adsorbed state is called the adsorbate [96]. The principle of the measurement consists of exposing the dry sample to a gas or vapour, under constant temperature condition, which is chosen for practical reasons, as the boiling point of the gas [97]. The adsorbed amount is then calculated from the pressure difference before and after equilibration or by weighing, in the case of the water adsorption method [82,98]. The measurement of the amount of gas adsorbed over a range of stepwise increasing partial pressure is known as an adsorption isotherm, while desorption isotherms are achieved by measuring gas removed as pressure is reduced.

Depending on the gas type, adsorption allows the evaluation of pore sizes from about 0.35 nm up to 100 nm. Taking into account narrow spatial distribution, users usually adopt the pore size terminology, according to the classification of the International Union of Pure and Applied Chemistry (IUPAC), in which micropores are defined as pores with a width not exceeding 2 nm, pores with diameters of more than 50 nm are classified as macropores, and pores of intermediate size are termed mesopores [98]. By analyzing the adsorption isotherms, it is possible to quantify the volume of adsorbed gas in meso- and micropores by evaluating the hysteresis of the adsorption and the desorption isotherm and analyzing the adsorption isotherm at a low partial pressure [99]. Additionally, the shape of the hysteresis loop, formed by the adsorption and desorption branch, allows qualitative interpretation of the pore shapes [98]. The calculation of the total pore volume and the mean pore diameter can be determined from the amount of vapour adsorbed at a relative pressure close to unity, by assuming that the pores are filled with the adsorbate in the bulk liquid state [100,101]. In the case of pore-size distribution, measured by the N_2_ adsorption method, the Barrett-Joyner-Halenda (BJH) theoretical model, with the assumption of cylindrical pores, is the most commonly used [102,103], while advanced DFT and grand canonical Monte Carlo (GCMC) models have been developed for calculating the pore-size distribution from the CO_2_ adsorption isotherm [104,105,106].

Nitrogen (at 77 K) is the most commonly used adsorptive to characterize the surface area of mesopores of 2–50 nm and macropores below 100 nm in diameter [102]. However, a small quadrupole moment of nitrogen molecule can give specific, adverse interaction in small micropores, various surface functional groups and exposed ions; therefore, some researchers recommend the use of Ar at 87 K [101,107]. In turn, the CO_2_ method is frequently used to measure the structure of micropores and narrow constrictions with widths smaller than 2 nm, due to the greater kinetic energy of the molecules and speeding diffusion into the narrow pores [104,108,109]. Other alternatives for N_2_ method include krypton (at 77 K) [110], helium (at 4.2 K) [111], or the adsorption of polar liquids, such as water vapour (at 293 K) [112]. The use of water vapour allows for the assessment of the so-called internal surface area, pore size distribution, and the interaction with polar surface functional groups that constitute adsorption sites for water vapour molecules [113,114,115]. However, in the case of some porous materials, such as clay minerals, the irreversible modification of the pore structure, due to the addition of water, is highly probable and lead to erroneous results [70,71,116].

Methods based on gas sorption are non-destructive, present medium repeatability of measurements, and can take from few hours to days [82]. Similar to MIP method, they cannot measure closed porosity and require the sample surface to be dry and cleansed of other gases prior to analysis [117]. To avoid measurement errors, due to sample pretreatment, an adequate combination of low vacuum with temperatures should be used [118]. Outgassing at too a high temperature or under ultra-high vacuum conditions may lead to changes in the surface composition, such as decomposition of hydroxides or carbonates and the formation of surface defects or irreversible changes in texture [109,117].

Methods for determining porosity, based on the observation of radiation changes, are a large part of modern non-destructive techniques. These include (among others): small and ultra-small angle neutron scattering (SANS and USANS), and small and ultra-small angle X-ray scattering (SAXS and USAXS), computed microtomography (CMT), nuclear magnetic resonance (NMR), as well as X-Ray, optical, AFM, TEM, SEM, FEM, and ESEM microscopy [119,120,121,122]. SAXS and SANS measurements, especially combined with ultra SAXS or ultra-SANS and with a proper contrast matching technique, provide information on total porosity, with differentiation to open and closed pores [119,123], as well as on the distribution of pore sizes, pore morphology, or fractal nature of pore matrix interfaces. These methods enable the observation of objects from about 1 nm (10 Å) to about 1μm (200,000 Å) [124]. In the case of the above analyses, however, some problems are recognized with the inaccessibility of pores to given fluids, due to a non-wettable surface. Variation in the inaccessible pores may occur during the application of different fluid-like substances, for example H_2_O or toluene and their deuterated forms. Past studies try to resolve this problem by using non-adsorbing or weakly adsorbing gases, such as CO_2_ or deuterated methane (CD_4_) [125]. Some researchers also recommend a combination of different techniques to improve the accuracy of porosity description. Mergia et al. [119] have reported that linking USANS, nitrogen adsorption, and helium pycnometry is necessary for porosity determination of doped graphite, due to porosity transformation from open to closed. They also showed that for total porosity, both He pycnometry and USANS need to be employed. Calo et al. [126] used combined SAXS/TGA to improve the resolution of porosity development in carbons. SAXS can be additionally linked the with CMT imaging technique that provides access to volume-resolved information [127] and the extension of the measurement range to micrometer levels [128]. Thus, SAXS-CMT method offers information from high and low-resolution techniques linked to a three-dimensional image. This combination is well recognized in the study of rigid samples, like bones and teeth; however, analysis of soft matter, like tissues, is still challenging, due to the poorly organized structure of high heterogeneity [127].

The CMT belongs to a technique widely utilized in the study of pores: their nature, size, density, and shape, and thus, in assessing uncovering faults of materials [129,130]. Spatial representation of the internal structure is the most valuable benefit of this method as compared to traditional analyses [131]. Moreover, CMT shows potential for analysis of undesired contamination which was indicated in studies of Sinico et al. [132], who found that content of 0.14 vol. % of W in the CN2-CuCr1 sample can promote lack-of-fusion pores, due to insolubility of W with Cu. Some studies on porosity revealed that the CMT method can be considered as faster and more accurate, as compared to conventional techniques [133], but another one, showed that surface porosity derived from microscopic observation and CMT at the highest resolution could be without significant differences [131]. CMT porosity of materials was also found to be higher than expected, due to aggregates exclusion [134]. In general, the CMT method is fraught with some difficulties, considering high accuracy. Uncertainty of the results can result from the user or algorithm-defined value of threshold in image segmentation, beam fluctuations or hardening, and off-focal radiation [135]. The spatial resolution of CMT images is limited by X-ray energy, the number of pixels on the detector, and the diameter of the X-ray source. Hermanek and Carmignato [136], in studies on reference object with artificial defects, emphasized that voltage and current affect porosity, particularly for small defects. Nehler et al. [135], in studies of sandstones, found that tomographic pore-volume quantification is imprecise, due to partly unresolved structures as compared to SEM images. Differences in linear attenuation and material porosity are influenced by the different composition of materials [137]. Some solutions to these problems were proposed in the latest studies, by reducing noise and artifacts [138], proper selection of algorithms to porosity segmentation [139], correcting off-focus or secondary X-rays [140], and compensation nonlinear beam hardening [141]. Soulaine et al. [142] compensated, to some extent, limitations occurring even in the most advanced micro-CT devices by using a model approach for sub-resolution porosity. Improved contrast was obtained by Shoukroun et al. [143] by using Edge Illumination X-ray Phase Contrast Imaging (EI XPCi), a novel method.

Microscopy techniques are wide group of techniques used in the study of topography and porosity, when the exact position of a given pore or crack can be located, and no models or further assumptions are required [144]. The resolution of optical microscopy is in the order of 200 nm. According to the widely discussed classification, performed by Stefanidou [145], a stereo binocular microscope with a typical magnification of 8–40× can find pores with a diameter higher than 10 um. A better magnification level is offered by petrologic microscopy; however, artefacts related to preparing thin sections can limit the use of this technique. The pore system, in this case, can be highlighted by impregnation with epoxy, normal blue or rhodamine-B [74]. SEM microscopy brings possibilities for fast observations of pores from nanometers to millimeters. The method can be applied to the porosity measurement of various surface layers [146]. In this method, however, sample preparation is of key importance. Grinding and polishing may cause a risk to destroy the structure and bring artificial pores and fractures [145]. Argon ion milling can reduce this problem [147]. Moreover, samples should be degassed, dried, and, in some cases, covered with a thin layer of conductive gold or carbon [148]. The application of environmental SEM (ESEM) can reduce these problems to some extent [149]. Transmission electron microscopy could have excellent resolving power—even better than 1 nm, but the image volume is strongly restricted to low values (low sample representativeness) and ultramicrotome use can be needed [123,150]. AFM is an optimal technique for the study of the porosity of organic materials and minerals on the level of nanopores [74]. The studies of Arzate-Vázquez et al. [151] on chicken eggshell showed that a combination of this method with image processing could be suitable for obtaining roughness parameters, surface area, and pore size, as well as shape and pore density. Liu et al. [152], in their research on coal and coal-bearing shale, have reported good accordance of AFM surface topographies with SEM results; however, a limitation of this technique can be fixed image size.

The non-destructive method is also NMR. The possibilities of this method largely depend on magnetic field strength, an increase of which improves signal-to-noise ratio but can also distort magnetic field in materials saturated with liquids [74]. Some problematic issues can be the presence of clay minerals, as well as the detection of signals from micro-pores, due to the limitation of echo spacing [153,154], have reported high accuracy of NMR in porosity determination; however, permeability needed to be corrected by model modifications. Ultrasonic studies can be used to detect porosity; however, extraction of information on size or origin is rather difficult in this case [136,139]. Other methods, such as thermoporometry, inverse size exclusion chromatography, positron annihilation spectroscopy, muon spin resonance, or terahertz time-domain spectroscopy (THz-TDS), coupled with an index-matching medium, are less frequently used to test porosity, and their application, adaptation, or modification is most often due to the specific nature of the material.

Interestingly, studies comparing porosity from different methods show various relationships between results for particular samples showing both low, weak, and high data similarity [155,156,157]. Underestimation or overestimation shows the importance of the appropriate selection of the method and its parameters for the given research material. According to Hossen et al. [158], saturation methods (here saturation with silicon oil) are less complicated and cheaper for low-density materials like foams fluid than mercury porosimetry, while geometric method, SEM and BJH sorption methods can give, in this case, deviated results from expected ones. Studies by Ghasemi-Mobarakeh et al. [146] showed that surface layers of nanofiber materials should not be analyzed by mercury porosimetry or density measurements, due to the possible influence of high pressure. Opaque samples are not proper for light scattering based methods, but rather for SAXS or SANS [144].

## 6. Conclusions

The porosity of materials is an important factor in biomaterials research. The proper assessment of pores inside the material plays an important role for its future application. Thus, detailed analysis is necessary before undertaking any investigations.

The selection of an appropriate method of porosity assessment should take into account the specificity of the studied biomaterial: its mineralogical composition, crystallinity, physical form, and chemical properties, as well as its thermal, radiation, and mechanical resistance. Finding an ideal method for porosity investigation is particularly difficult for materials that are a mixture of organic and inorganic compounds.

Research indicates that the most comprehensive approach to examining such materials should involve an analysis using several complementary methods, covering the widest possible range of pore sizes. In addition to the constantly observed increase in the resolution capabilities of devices, the development of computational models and algorithms, improving the quality of the measurement signal, remains a big challenge.

## Figures and Tables

**Table 1:** Classifications of pores based on pores width and functions.

	Type of Pores, [nm]					
**Classification by Pore Size**	**Macro-**	**Meso-**	**Micro-**	**Supermicro-**	**Ultramicro-**	**Submicro-**
Dubinin, 1979	d > 200–400	200–400 > d > 3- 3.2	d < 0.6–0.7	3–3.2 < d < 1.2–1.4	-	-
IUPAC, 1972	d > 50	2–50	d < 2	0.7–2	d < 0.7	
Cheremskoj, 1985	> 2000	-	2000 > d > 200	-	<2–4	<200
**Classification by Pore Functions**	**Transmission**	**Storage**	**Residual**	**Bonding**		
Greenland and Pereira, 1977	50,000–500,000	500–50,000	d < 500	d < 5		

Bold headings indicate different pore classifications.

**Table 2:** Selected properties of various dental ceramics.

Properties	Dental Ceramics					
	Feldspar Ceramic	Leucite- Reinforced Glass Ceramic	Lithium Disilicate Ceramic	Fluorapatite Glass-Ceramic	Alumina Ceramic	Zirconium Oxide Ceramic
Flexural strength (MPa)	60–110	120–160	350–400	90–110	350–600	840–1200
Hardness by Vickers (GPa)	>6.5	6.67	5.3	4.5	11.5	13.7
Young’s modulus (GPa)	-	65	103	70	380	210
Density (g/cm^3^)	2.1	2.5	2.47	2.56	3.96	6.1
